# Formation of nanostructured silicon surfaces by stain etching

**DOI:** 10.1186/1556-276X-9-482

**Published:** 2014-09-11

**Authors:** Maha Ayat, Samia Belhousse, Luca Boarino, Noureddine Gabouze, Rabah Boukherroub, Mohamed Kechouane

**Affiliations:** 1 Centre de Recherche en Technologie des Semi-conducteurs pour l'Energétique (CRTSE), Thin Films, Surface and Interface Division, 02, Bd. Dr. Frantz Fanon, B.P. 140, Alger-7 Merveilles, 16038 Algiers, Algeria; 2 NanoFacility, Instituto Nazionale di Ricerca Metrologica, Strada delle Cacce 91, Torino 10135, Italy; 3 Université des Sciences et Technologies Houari Boumediene (USTHB), B.P. 32, El Alia, Bab Ezzouar, 16111 Algiers, Algeria; 4 Interdisciplinary Research Institute (IRI), IRI-IEMN, Avenue Poincaré, B.P. 69, 59652 Villeneuve-d'Ascq, France

**Keywords:** Silicon, Etching time, Vanadium oxide, Catalyst

## Abstract

In this work, we report the fabrication of ordered silicon structures by chemical etching of silicon in vanadium oxide (V_2_O_5_)/hydrofluoric acid (HF) solution. The effects of the different etching parameters including the solution concentration, temperature, and the presence of metal catalyst film deposition (Pd) on the morphologies and reflective properties of the etched Si surfaces were studied. Scanning electron microscopy (SEM) was carried out to explore the morphologies of the etched surfaces with and without the presence of catalyst. In this case, the attack on the surfaces with a palladium deposit begins by creating uniform circular pores on silicon in which we distinguish the formation of pyramidal structures of silicon. Fourier transform infrared spectroscopy (FTIR) demonstrates that the surfaces are H-terminated. A UV-Vis-NIR spectrophotometer was used to study the reflectance of the structures obtained. A reflectance of 2.21% from the etched Si surfaces in the wavelength range of 400 to 1,000 nm was obtained after 120 min of etching while it is of 4.33% from the Pd/Si surfaces etched for 15 min.

## Background

In the current semiconductor industry, nanostructures of silicon represent the basic material for the conception of several devices in the field of nanoelectronics [1,2], optoelectronics [3], energy conversion [4,5], energy storage [6,7], and also (bio)chemical sensors [8,9]. Various methods have been developed to fabricate Si nanostructures such as reactive-ion etching (RIE), electrochemical etching, metal-assisted etching, or stain etching. This last one is an electroless method of forming porous silicon (PSi) in a mixture based on hydrofluoric acid (HF) and an oxidant. The nanostructuring of silicon by stain etching has attracted increasing attention in recent years for several reasons. One of these reasons is that it is an inexpensive method with the ability to control various parameters and can be accomplished in a simple chemical laboratory. The most widely used oxidant is nitric acid (HNO_3_) which has been investigated in numerous studies for the development of silicon nanostructures (nanopillars and nanowires) for their interesting fields of application [10]. However, the use of HNO_3_ leads to bubble formation, inhomogeneous films, and irreproducible results.

Recently, Kolasinski and Barclay demonstrated that using vanadium oxide (V_2_O_5_) in the etching solution seems to be a good way to avoid bubble formation (no gas is generated). V_2_O_5_ dissolves in HF(aq) to form VO_2_^+^, which according to the half reaction $VO_{2}^{+}+H^{+}+e^{-}\to VO^{2+}+OH^{-}$ with an appropriate acceptor level at *E*_0_ = +1.0 V, which is able to inject holes into the Si valence band [11]. Several studies on the kinetics of the stain etching using V_2_O_5_ have been demonstrated, but few works on the morphological properties of the structures have been obtained.

In this work, we report the fabrication of ordered array pillar silicon and silicon macropores by a simple chemical etching of silicon in vanadium (V) oxide/hydrofluoric acid solution. Different etching parameters including the solution concentration, temperature of Si substrates, and thin metal catalyst film deposition (Pd) on the Si surface were studied. The etched surfaces were characterized by scanning electron microscopy and spectrophotometry.

## Methods

Twenty-nanometer palladium (Pd) films are deposited by evaporation technique, on one side of p-type (1 to 10 Ωcm) single crystal (1 0 0) silicon wafers (both sides polished), with 250 to 300 μm thickness, cleaned using a dilute HF aqueous solution (1:10) for 30 s prior to deposition. Deposition of a significantly thinner Pd film can result in uniform etching of the entire silicon surface during chemical etching. The last step of the fabrication process consists of etching the Si and the Pd/Si samples in a mixture of HF (49%; Sigma-Aldrich, St. Louis, MO, USA) and V_2_O_5_ (98%; Sigma-Aldrich), for a period of 30, 90, and 120 min. Only one side is in contact with the etchant by using an adapted cell, and the edges are protected by an O ring.

After etching, the samples are thoroughly rinsed in DI water and dried with nitrogen stream. The etch rate was obtained by dividing the etch amount, i.e., Si weight before and after the etching process of Si by reaction time, exposed surface area, and Si density (2.33 × 10^3^ kg/m^3^). Photoluminescence analyses were performed with a PerkinElmer LS 50B (PerkinElmer, Waltham, MA, USA) spectrometer.

The UV-Visible reflectance of the etched silicon was measured using a Cary 500 Varian spectrophotometer (Varian Medical Systems Inc., Palo Alto, CA, USA) in the range of 400 to 1,100 nm. The surface morphology and microstructure of the etched silicon surface were investigated using JEOL JSM 6360 LV (JEOL Ltd., Akishima-shi, Japan) scanning electron microscope (SEM) equipped with energy dispersive X-ray spectroscopy (EDS) and a FEI Inspect F-SEM (FEI, Hillsboro, OR, USA) equipped with FEG (field emission gun). The Fourier transform infrared spectrometry (FTIR) was performed with a Thermo Nicolet spectrometer (Thermo Fisher Scientific, Waltham, MA, USA). IR spectra were recorded at a resolution of 4 cm^-1^ by averaging 32 scans.

The presence of Pd traces during the etching was performed using secondary ion mass spectroscopy (SIMS) 4E7.

## Results and discussion

**Figure 1:**
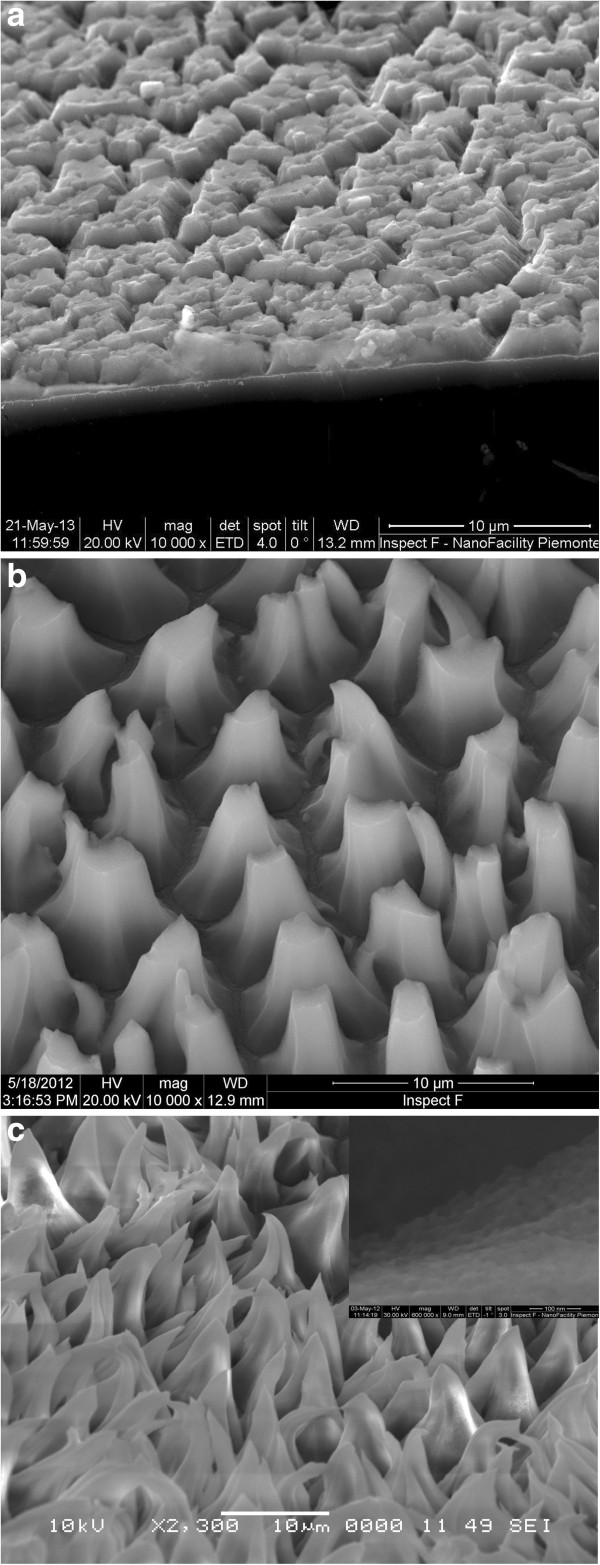
**SEM images for silicon substrate etched in HF/V**_**2**_**O**_**5 **_**solution at different times. (a) 30, (b) 60, and (c) 120 min.**

The morphology of the Si etched surface was examined by SEM. Figure 1a,b,c shows SEM images of silicon surface etched in V_2_O_5_ solution at different times, 30, 60, and 120 min. For an etching time of 30 min (Figure 1a), the formation of nanoporous silicon layer appears in the form of islands separated by large channels, which resembles the electrochemically etched n-type silicon surface in HF/ethanol solution [12,13]. Increasing the etching time to 60 min (Figure 1b) leads to the formation of pyramidal or pillar structures with a flat summit. These pillar structures become rounded facet with a curved and sharp summit at an etching time of 120 min (Figure 1c). The height of the pillar measured by SEM is about 10 μm.

The obtained morphologies are found to be different from those generally observed with other oxidants [14,15], such as H_2_O_2_, K_2_CrO_7_, HNO_3_, where different structures have been obtained. For example, it was shown that etching of Si substrate in HF/H_2_O_2_ solutions leads to the formation of nanoporous and macroporous structures. The stain etching can produce both PSi and silicon nanowires (SiNW). The hydrogen terminated surface (H-Si) is important in the formation of both structures. The obtained morphologies are probably due to the particularity of using V_2_O_5_ which dissolves in HF to form ${\mathrm{VO}}_{2}^{+}$ (one hole is injected) which is reduced to VO^2+^ during PSi nanostructure formation. Indeed, the discovery of stain etching with V_2_O_5_ as oxidant by Kolasinski et al. [16] has provided a unique opportunity to study the stoichiometry of stain etching. Moreover, Kolasinski et al. [16] have explained the simultaneous doubling of ${\mathrm{VO}}_{2}^{+}$ consumption and 50% reduction in H_2_ evolution by etching according to the following reaction:

(1) $$\begin{array}{l} \mathrm{Si}\;+\;2\mathrm{HF}\;+\;2{\mathrm{HF}}_{2}^{‒}\;+\;{\mathrm{VO}}_{2}^{+}\to {\mathrm{SiF}}_{6}^{2‒}+\;H_{2}\\ \;+\;{\mathrm{VO}}^{2+}+\;H_{2}O\;+\;e_{\mathrm{cb}}^{‒} \end{array}$$

This reaction is accompanied by a charge-balancing counter reaction

(2) $${\mathrm{VO}}_{2}^{+}\;+\;2H^{+}\;+\;e_{\mathrm{cb}}^{‒}\to {\mathrm{VO}}^{2+}+\;H_{2}O$$

**Figure 2:**
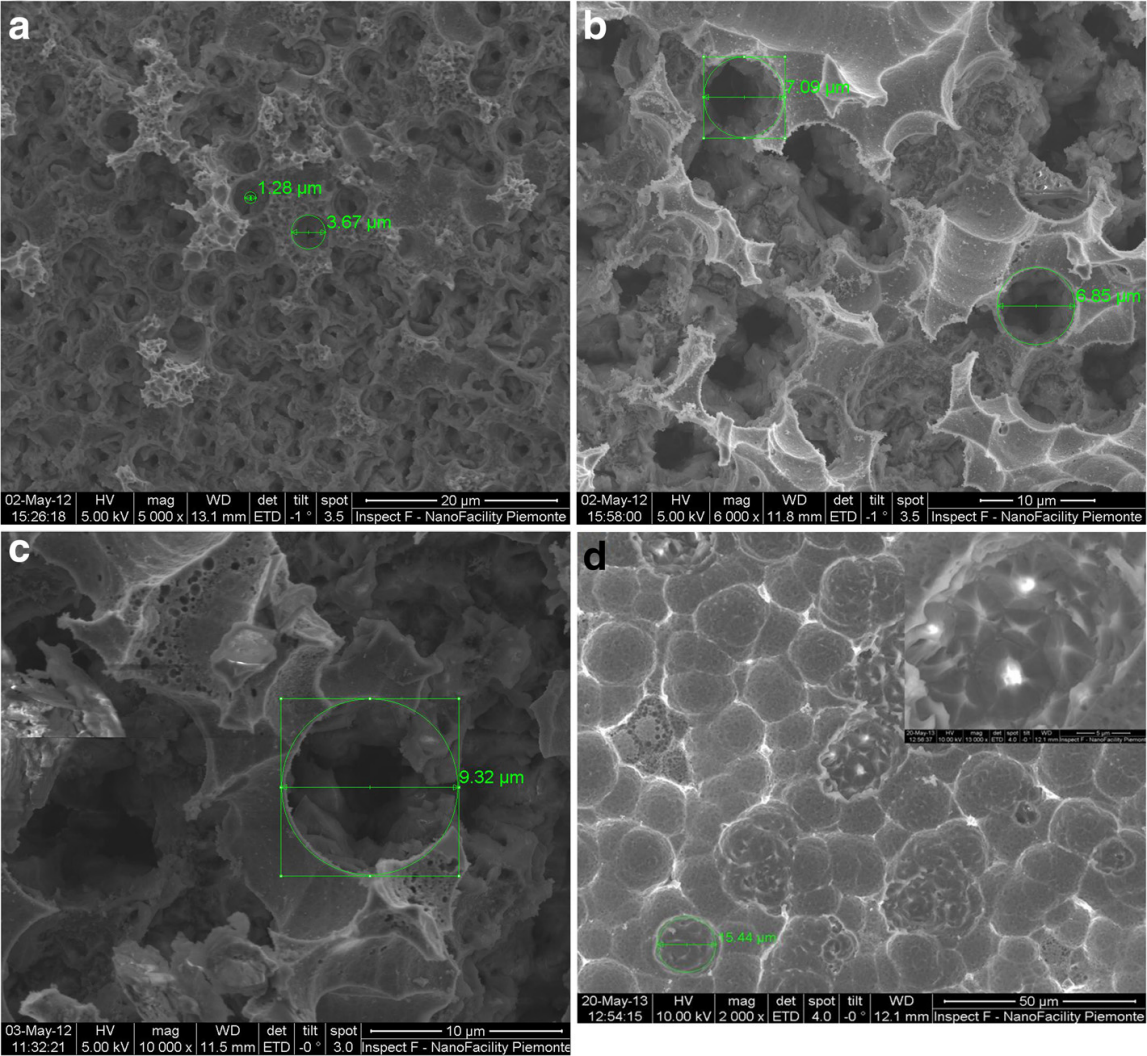
**SEM images for Si/Pd substrate etched in HF/V**_**2**_**O**_**5 **_**solution for different times. (a)** 30, **(b)** 60, **(c)** 90, and **(d)** 120 min.

**Figure 3:**
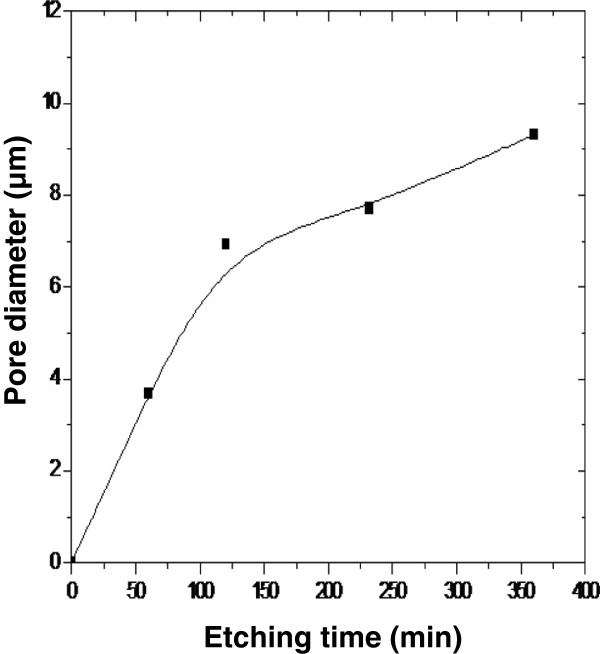
**Variation of pore diameter of Pd/Si samples etched in HF/V**_**2**_**O**_**5.**_

Figure 2a,b,c,d shows SEM images of Pd-coated Si subjected to etching solution for 30, 60, 90, and 120 min where the presence of circular pores on the surface is revealed. In addition, it is shown that the pore size evolves with the increase of etching time (Figure 2). Pore diameter increases rapidly for an etching time of 60 min, to reach a value of about 7 μm, and then increases slowly for etching time higher than 120 min. A value of 9 μm is measured for an etching time of 350 min as depicted in Figure 3. This behavior could indicate that the catalyst effect of Pd becomes less predominant as the chemical reaction time increases.Indeed, the amount of Pd deposited on silicon surface reacts and dissolves in the etching solution leading to the diminishing of the concentration of holes on the silicon surface and then of the etching rate. This chemical etching leads to the etching in the interior of macropores and formation of pyramidal structures. The increase in the etching time favors the formation of new structures inside the macropores. As shown in the inset of Figure 2d, as the Pd effect disappears, the pore wall will be broken by the etching solution and new pyramidal structures appear.

The widening of the pores could be due to different reasons: (1) Si without metal is etched as well due to the diffusion of injected holes from the etching front to the side of the pore wall [17,18], and (2) Si without Pd metal coverage is etched slowly in the etchant [19].

**Figure 4:**
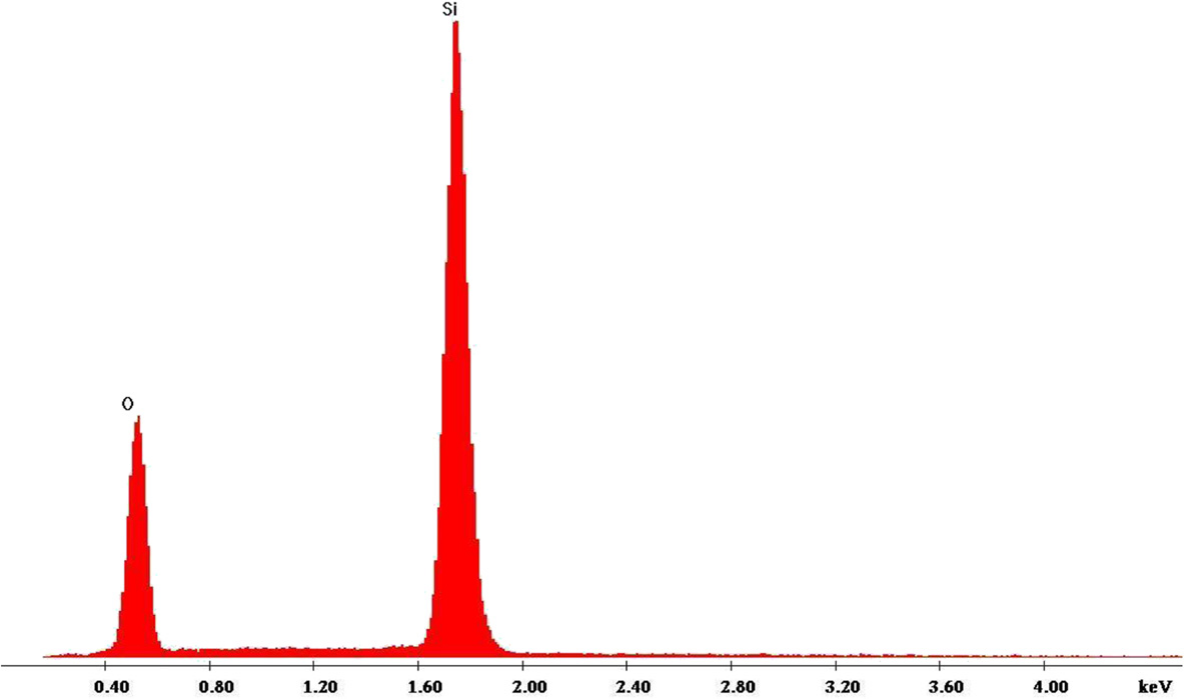
**EDS spectrum of Si/Pd substrate etched in HF/V**_**2**_**O**_**5 **_**for 120 min.**

**Figure 5:**
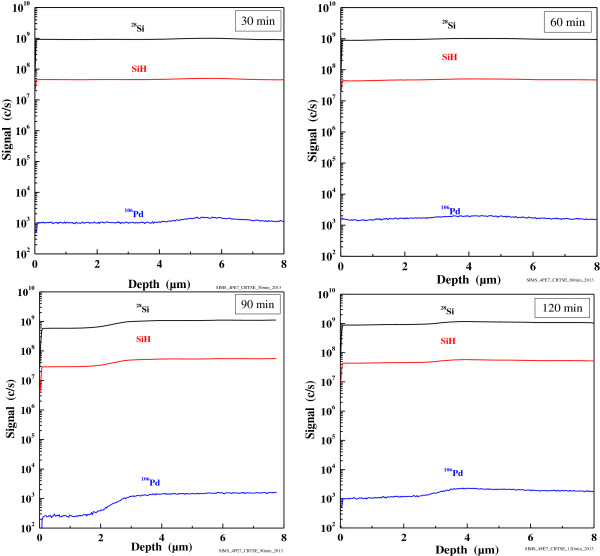
**SIMS curves of Si/Pd samples etched in HF/V**_**2**_**O**_**5 **_**for different etching times.**

Elemental analysis by EDS of Pd-coated Si surface subjected to the etching solution for 120 min (Figure 4) revealed that the Si surface is mainly composed of Si and a bit of oxygen, probably due to oxidation of the Si sample. One can note the absence of Pd on Si surface, indicating its consummation during the chemical etching. SIMS characterization performed on Pd/Si etched samples for different times (30 to 120 min) confirms the absence of palladium traces into the samples at 9 μm from the top of the surface, as shown in Figure 5. We can observe that the Pd signal is under the detection limit (10^4^ to 10^5^ c/s).

Etching time effect on the rate was examined by measuring the etch depth as a function of etching time in the range of 0 to 360 min. The etch depth was also compared with that measured by SEM.

**Figure 6:**
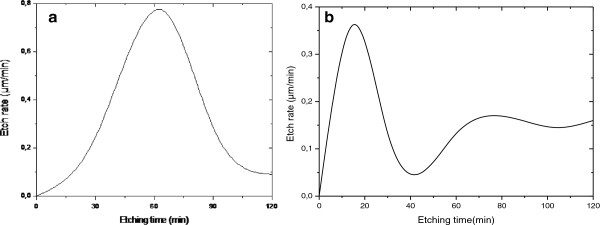
**Variation of the etch rate of silicon (a) and Si/Pd samples (b) etched in HF/V**_**2**_**O**_**5 **_**(0.12 M).**

Figure 6a shows the measured etch rate of Si samples etched in HF/V_2_O_5_ (0.12 M). It is seen that the etch rate increases rapidly with etching time, reaches a maximum at 60 min, and then decreases. The diminishing in the etch rate is due to the decrease of vanadium concentration in the solution.

Figure 6b corresponds to the etching of Pd-coated Si in solution as previously mentioned. It shows that the etch depth first increases linearly with time, reaches a maximum at about 20 min, then decreases to attain a minimum etch rate value for 40 min, and finally increases to stabilize at an etching time of 70 min. This behavior is interesting since it confirms that the etching of Pd-coated Si samples in HF/V_2_O_5_ occurs in two steps. In the first step, the metal-assisted chemical etching is predominant (Pd catalyst effect), and in the second step, the stain etch is involved (Pd is consumed in the etching).

**Figure 7:**
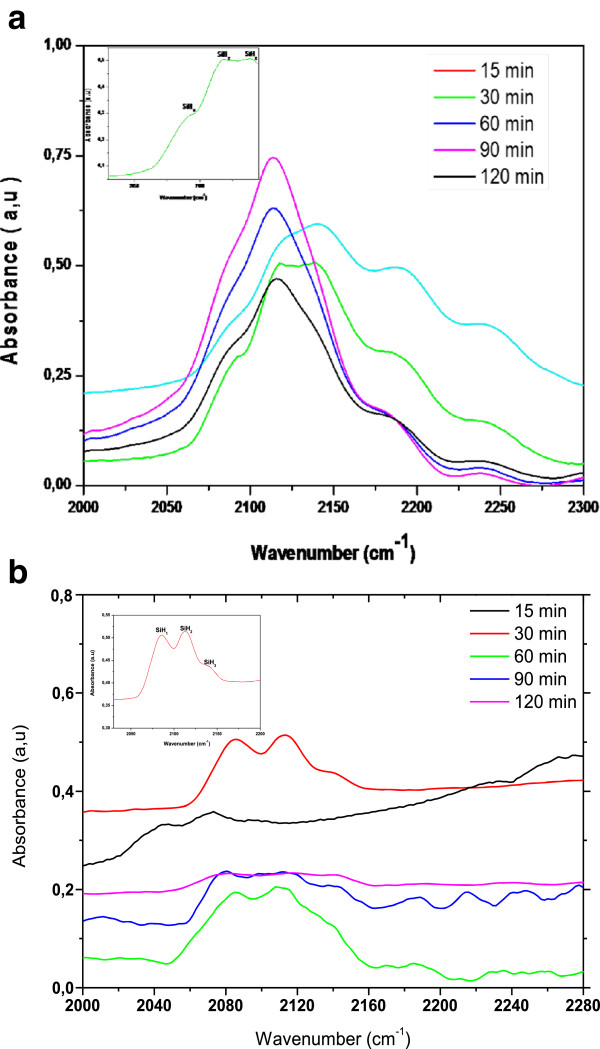
**FTIR spectra of Si (a) and Pd/Si samples (b) etched in HF/V**_**2**_**O**_**5 **_**(0.12 M) for different etching time.**

FTIR measurements of Si and Pd/Si etched samples in HF/V_2_O_5_ solution are discussed in the following section. Etched Si samples in HF/V_2_O_5_ reveal the presence of hydride (SiH_x_) species on the surface (Figure 7a). A shouldering, associated with monohydride (SiH), appears at 2,081 cm^-1^. The spectrum is dominated by pronounced peaks at 2,113 cm^-1^, associated with dihydride (SiH_2_) species, which becomes larger over etching time.

**Figure 8:**
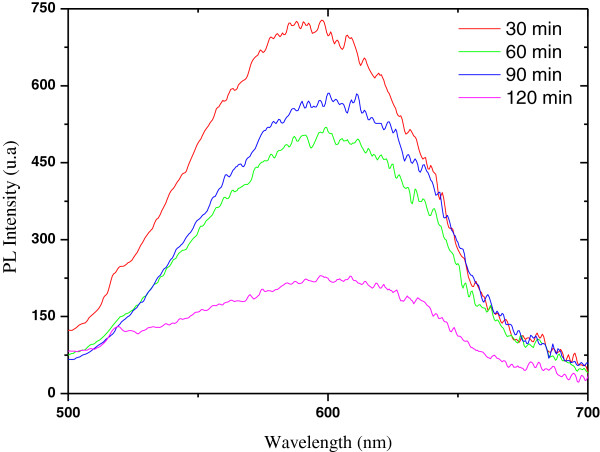
**Photoluminescence spectra of Si samples etched in HF/V**_**2**_**O**_**5 **_**for different etching times.**

For the Pd/Si etched films (Figure 7b), the surfaces are H-terminated. It has been shown that the Si-H bonds evolve with etching time. At an etching time of 15 min, the formation of Si-H bonds appears. We note also the presence of peaks at 2,188, 2,215, 2,245 cm^-1^ indicating that O atoms are inserted in Si-H surface bonds during the second part of the etching. The formation of these SiH_x_O_y_ bands is observed at longer etching time, probably caused by oxidation induced by water during the PSi formation.Figure 8 shows photoluminescence (PL) spectra dependence on etching time. We observe a red shift and a decrease of PL intensity with increasing etching time. The maximum PL intensity is found for an etching time of 30 min at 590 nm, while the etched layer for 120 min exhibits luminescence with a peak at 610 nm. It is interesting to notice that the peak intensity can be easily controlled by the etching time.

The decrease of PL intensity with increasing time is probably due to the reduction of nanostructure (nanocrystallites) density.

**Figure 9:**
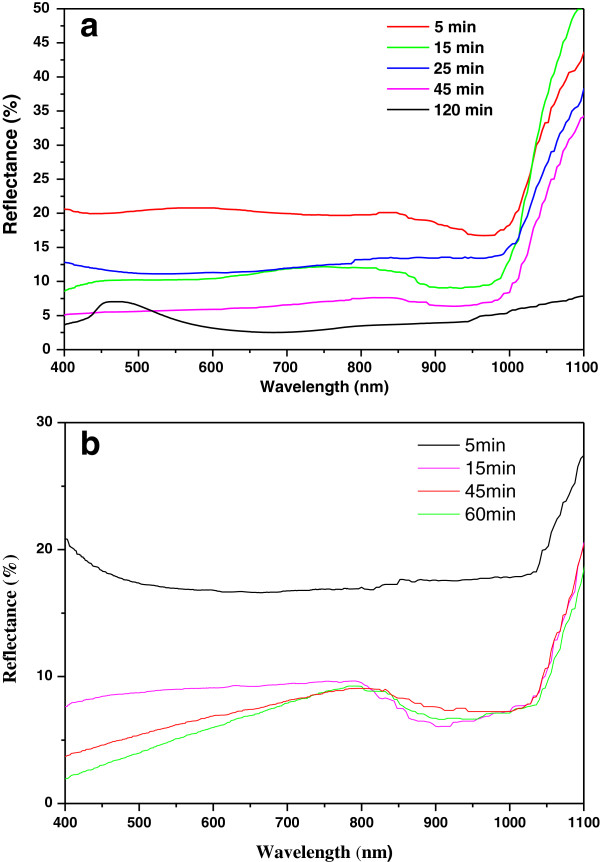
**Reflectance curves of etched samples in HF/V**_**2**_**O**_**5 **_**(0.12 M) at different etching times. (a)** Si samples and **(b)** Pd/Si samples.

The reflective properties of the samples etched at different conditions in HF/V_2_O_5_ solution are shown in Figure 9a,b. It is shown that Si etched for 120 min presents a low mean reflectance of 2.21% in the wavelength range of 400 to 1,000 nm. This can be explained by the uniform pyramidal structures shown in Figure 1c. The structures obtained at short etching time show a high reflectance probably due to the densification of the structure. Whereas for the Pd/Si samples etched in the same conditions, as previously mentioned, the mean reflectance increases as the etching time increases. A mean reflectance value of 4.33%, in the wavelength range of 400 to 1,000 nm, is obtained for an etching time of 15 min. The results demonstrate that these etching methods reduce the surface reflectance to values less than 4.33%. Thus, they can be used in the fabrication process of photovoltaic cells.

## Conclusions

The results show that Si etching in HF/V_2_O_5_ solution induces the formation of pyramidal or pillar structures which evolve with etching time. The presence of metal catalyst (Pd) does not only accelerate the etch rate but also creates pyramidal structures within macropores whose diameter increases with etching time till the total consummation and disappearance of the Pd film. A low mean reflectance value of 2.21% in the wavelength range of 400 to 1,000 nm was observed for Si samples etched in V_2_O_5_/HF for 120 min, while a mean reflectance value of 4.33% was obtained by etching the Si/Pd samples in the same conditions for 15 min. Finally, the reflectance spectra of both Si and Si/Pd surfaces indicate that low mean reflectance is obtained which suggest a potential application in photovoltaic solar cells.

## Competing interests

The authors declare that they have no competing interests.

## Authors' contributions

MA performed the experiments and drafted the manuscript. LB carried out the SEM analyses of the obtained structures and helped in drafting the present manuscript. SB participated in the interpretation of the obtained FTIR spectra and in revising the manuscript before the final submission. RB introduced the study of stain etching of silicon and participated in the revision of the manuscript. NG supervised the study and helped in writing the manuscript. MK participated in the revision of the present manuscript and added some new ideas. All authors read and approved the final manuscript.
